# EDITORIAL

**DOI:** 10.1002/reg2.7

**Published:** 2014-01-16

**Authors:** Susan V. Bryant

**Affiliations:** ^1^ School of Biological SciencesUniversity of California IrvineIrvineCA92697USA

I am both honored and excited to be announcing the launch of *Regeneration*, the first ever major journal dedicated exclusively to research on regeneration and repair mechanisms in animals and plants. My goal as Editor‐in‐Chief is for *Regeneration* to emerge quickly as the venue of choice for publication of research on regeneration by the large community of scientists whose works range from appendage and organ regeneration in species from across the evolutionary spectrum, to stem cells and the *in vitro* creation of organs with multiple cell types. We launch *Regeneration* as an open access journal with an experienced Managing Editor, Lisa Hannan, an internationally stellar group of Associate Editors, and members of the Editorial Board representing the breadth and depth of the field. We are indebted to our colleagues Kiyo Agata and Wiley Editor Pernille Hammelsoe for the initial impetus to create this new and important venue for expanding the field of regeneration.

Throughout the history of regeneration research, there has always been the hope that what we learn from regenerating animals will lead eventually to improved healing and regeneration of form and function for humans. The combination of the fields of developmental, regenerative and stem cell biology is providing an avenue for organ development *in vitro*, as recently shown by the successful development of an eye from mouse stem cells in a dish. The need for a better understanding of the guiding cues and cellular responses by which cells form tissues and tissues form organs is starting to be appreciated; and at the same time, the basic molecular and cellular tools needed for these next steps are now in place.

The launch of a new journal devoted to regeneration reignites the hope of regeneration, but this time with a difference, because now we have tools, technologies and insights emerging from the rapidly advancing new fields of genomics and stem cell biology. Genomics is opening up a treasure trove of new and unanticipated information about how genes are regulated, leading to a possible future in which reagents will be developed that are capable of controlling tissue‐specific repair processes. The rapidly developing stem cell field has already shown that stem cells have the potential to ameliorate the negative consequences of a number of degenerative disease conditions.

The next major challenge on the horizon is not just patching up damaged organs but the complete regeneration of damaged or missing organs and appendages. Understanding the way in which both animals and plants are able to accomplish complete, total and repeated regeneration of body parts is an essential first step. Humans are unable to match the regenerative feats of our close vertebrate relatives who share the same basic body design and developmental processes. Some amphibians can regenerate arms and legs as well as many other organs, whereas humans can only regenerate components of these organs (bone and muscle and skin), not the structures that are composed of these tissues.

During the next decade, much attention will probably be focused on why regeneration‐competent animals can restore their missing body parts but mice and humans cannot. Already over the last decade we have witnessed a renewed interest in regeneration and the number of scientists using regeneration‐competent animal models to tease apart the molecular and cellular details of the process has increased. This work will accelerate over the next period of time, and future steps will include strategies to induce regenerative responses in the tissues and organs of non‐regenerating mammals, with eventual application to humans. New avenues of communication specific to the task at hand are essential and I would like to commend the efforts of our colleague Francesc Cebrià at the University of Barcelona whose Regeneration Blog (http://regenerationinnature.wordpress.com) provides a venue for discourse on this topic. Our new journal, *Regeneration*, will provide a novel platform for the exchange of a wide range of new findings relevant to regenerative biology that extends from basic biology to medical application and all steps in between, with the goal of realizing our own regenerative ability using our own cells.

